# Protective efficacy using Cape- golden berry against pre-carcinogenic aflatoxins induced in rats

**DOI:** 10.1016/j.toxrep.2019.06.012

**Published:** 2019-06-19

**Authors:** Ahmed Noah Badr, Mohamed Ahmed Naeem

**Affiliations:** 1 Food Toxicology and Contaminants Dept., National Research Centre, Dokki 12622, Cairo, Egypt; 2 Ain Shams Specialized Hospital, Ain Shams University, Cairo 16096, Egypt

**Keywords:** Aflatoxins, Biochemical parameters, Cape-golden berry, Carcinogenic impacts, Liver tissues, Corrective action

## Abstract

Aflatoxins are harmful compounds that induced carcinogenic impacts on tissues. It could generate oxidative stress causing cells damage. Bioactive substances from natural plants could avoid mycotoxins’ bad impacts. Cape-goldenberry (CGB), a source of active substances, was vacuum-dried at 30 °C then milled. Fresh and dried CGB-powder properties were estimated. Animal experiment was designed using six rat-groups to evaluate CBG effect to reduce harmful effect of aflatoxins. Rats treated groups were orally administrated by aflatoxins (AFs) with or without CGB in diets. Blood parameters, liver and kidney functions, serum lipids, and liver histological changes were estimated. The CGB powder showed several time doubles of phenolics, flavonoids, and antioxidants than fresh fruits. Diet supplementation by CGB of AFs-treated rats showed enhancement in final weight, food efficiency, and weight gain compared to AFs treatment only. Also, liver and kidney functions, liver enzymes, iron level, tumors indicator, and serum lipids of AFs- rats. Moreover, total protein, albumin, and globulin reduction by AFs have been improved by CGB presence in diets. Histopathological studies for AFs-rats liver showed dilated blood sinusoids with aggregation of inflammatory, Kupffer cell hyperplasia, degenerated hepatocytes, and apoptotic cells. However, in AFs-rat groups fed CGB in diets, liver hepatocytes appeared to be almost normal similar to the control. Results pointed out that CGB recorded a corrective action for aflatoxin B_1_ and G_1_ toxicity. This was recorded for the blood and serum parameters, and liver enzymes. This CGB action avoiding AFs-toxicity was more clearly declared in the liver tissues.

## Highlights

• Vacuum drying saves the CGB bioactive components.
• Addition of CGB to rats’ diet presents good health effects.
• Aflatoxins caused vigorous impacts for rats’ biochemical parameters and tissues.
• CGB showed an ability for AF–precarcinogenicity reduction in liver tissues.
• CGB recorded enhancing the liver enzymes and blood parameters of AFs-rats.

## Graphical abstract

**Figure:**
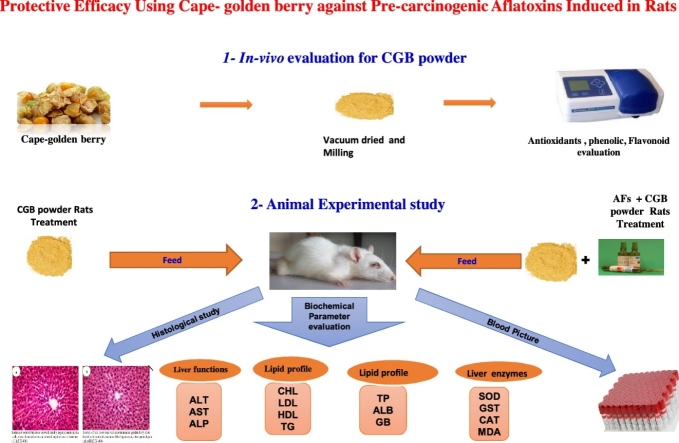

## 1 Introduction

Bioactive molecules from plants are important therapeutic agents for medicine due to their alternatively to chemical products and most of them have valuable bioactive phytochemicals compounds. Cape-goldenberry (CGB) is the common name of a plant, which is also called in many names such as Cape gooseberry or ground cherry [1,2]. It is also known in some places by other names such as: Chinese lantern or husk tomato. This plant is a member of the family *physalis*, a group of plant close to the tomato [3,4]. It is native to several places; however, it has grown in a wide area of the world either as a harvested plant or wildly grew. It is quite regarding great edible plants number, including tomato, courgette, green pepper, and eggplant [5].

The CGB is a plant with an economically beneficial and marketed in Egypt as a summer fruits ready to eat as an amusement food. These phytochemicals have physiological effects on humans due to the antioxidant, antibacterial, and antifungal activities of flavonoids, terpenoids, vitamins, and alkaloids. Antioxidants are substances that significantly prevent oxidation of an oxidizable substrate present at low concentrations [4]. The activities of free radicals involved in destruction of DNA, cancer, and aging [[6], [7], [8]].

Many compounds of the CGB plants have been isolated and identified as valuable drugs in modern medicine system [[9], [10], [11]]. Recently, CGB has a little historical treating disorder in the classical medicine [12]. Researchers still make an effort to screen plant natural components reducing apoptosis-induced proteins of cancer therapy in human cancers. For this reason, it is necessary to search for apoptosis-inducing novel compounds as postulant anticancer agents. There is an interest in exogenous antioxidants such as natural phenolics of plants might decrease oxidative damages without causing side effects [13]. The CGB hot air drying improved qualities of dietary fiber content [14]. The fruit showed a variation in polyphenols and vitamins content according to factors like harvesting time and ripening [15].

Aflatoxins (AFs) are exceedingly the highest hazardous health' enemies for human and animal, it can cause mutagenicity and carcinogenesis effects through feed and food contamination [[16], [17], [18]]. Many food materials contaminated by toxigenic fungi [[19], [20], [21]], which rise mycotoxin-excretion on food commodities [22,23]. In comparison to other contaminants, AFs are a great source to chronic diseases exposure like cancers particularly aflatoxin B_1_ (AFB_1_), related to mycotoxin presence in body fluids [16,[24], [25], [26]]. The present study aims to clarify the potential impact of CGB as anticancer agent against aflatoxin G_1_ (AFG_1_) and clear its ability to reduce the proportion of pre-carcinogenic compound represented by AFs in light of the bioactive substances involved in this plant.

## 2 Materials and methods

### 2.1 Materials

CGB fruits were purchased from Experimental Farm at Kom Hamada, Al-Buhayrah Governerate, Egypt during winter seasons 2017/2018. Intact goldenberry fruit were carefully selected according to the degree of ripeness measured by fruit color (brilliant orange). The standard of AFs received as dry films or crystals to container of dry AFs. Hemoglobin, serum iron, total iron binding capacity (TIBC), serum alanine amino-transferase (ALT), serum aspartate amino-transferase (AST), alkaline phosphatase (ALP), total protein (TP), albumin (ALB), globulin (GLB), urea, creatinine, uric acid, cholesterol (CHL), low density lipoprotein (LDL), high density lipoprotein (HDL), triglyceride (TG), total antioxidant (TAA), malondialdehyde (MDA), super oxide dismutase (SOD), glutathione-S-transeferase (GST) and catalase (CAT) were purchased from SPINREACT Co, SPAIN 2016.

### 2.2 Methods

#### 2.2.1 Preparation of concentrated powder

Fruits were sorted, de-hulled, washed, dried, and mixed using a hand mixer. Aluminum trays were utilized to make a slim layer of mixed fruits at 30 °C using the vacuum oven. The dried fruits of CGB was well grounded using Cyclone Mill Twister for well homogenizing followed by sieving through 60 mesh sieves, packed in easy-closed poly-ethylene bags, and stored under cooling till the application.

#### 2.2.2 Total phenolics content

The Folin–Ciocalteu reagent assay was used to determine the total phenolics content of extracts as described by Badr et al. [17]. The total phenolics content was expressed in mg Gallic acid equivalents (mg GAE)/g sample). All determinations were performed in triplicate.

#### 2.2.3 Total flavonoid content

The total flavonoids contents of the CGB was determined by a colorimetric method as described by Shehata et al. [18], the absorbance was measured at 510 nm. The results were expressed in mg Cat. /g sample. All determinations were performed in triplicates.

#### 2.2.4 Evaluation of antioxidant activity

##### 2.2.4.1 The DPPH radical scavenging activity

The free radical scavenging activity of CGB was measured by the DPPH method as proposed by Abdel-Razek et al. [27]. The measured conditions recorded at 517 nm.

##### 2.2.4.2 ABTS cation de-colorization assay

The ABTS radical assay was used to evaluate the ability to scavenge free ABTS radicals, based on the method applied by Badr et al. [17]. Absorbance readings were measured at 734 nm. Results were expressed as μ mol trolox equivalents (TE) /g sample from a standard curve developed with Trolox.

##### 2.2.4.3 Ferric reducing ability (FRAP) assay

The FRAP assay was done according to Hwang and Do-Thi [28]. The colored product absorbents were measured at 593 nm. The standard curve was prepared using Trolox, results were expressed as mM Torlox equivalent (TE/g sample).

#### 2.2.5 Preparation of standards for aflatoxin

A volume of methanol: acetonitrile (9:1) was calculated to give a concentration of aflatoxin as ng/ml.

#### 2.2.6 Experimental animals

One month old Albino male rats (140–150 g) were purchased from Animal House Unit, National Research Centre, Cairo, Egypt. Rats maintained on standard lab diet (protein: 160.4; fat: 36.3 and fiber 41 g/kg), the CGB dried powder was added to the basal diet components in treated groups at 20% (w/w) depending on previous *in vitro* work [29]. Rats were housed in a room free from any source of chemical contamination, artificially illuminated and thermally controlled. Animal procedures were performed in accordance with the Ethics Committee of the National Research Centre, Cairo, Egypt, and followed the recommendations of the National Institutes of Health Guide for Care and Use of Laboratory Animals (Publication No. 85-23, revised 1985).

#### 2.2.7 Experimental design

Animals were divided into six groups (G), each group had five rats housed individually in filter-top polycarbonate cages, and maintained on their respective extract for 35 days. Aflatoxin doses were injected to rats dissolved in phosphate buffer saline (PBS) at 850 ng/kg/b.wt./day as follow:

**Group (1):**Negative control fed on basal diet and water without any treatment

**Group (2):** Positive control fed on basal diet + CGB (AFs-free residues).

**Group (3):** Fed on basal diet + AFB_1_.

**Group (4):** Fed on basal diet + AFG_1_.

**Group (5**): Fed on basal diet + CGB + AFB_1_.

**Group (6):** Fed on basal diet + CGB + AFG_1_.

The AFs doses were used depending on our previous studies [29,30], animals were observed daily for signs of toxicity and weighted as well. At the end of experiment period, blood samples were collected from all animals from retro-orbital venous plexus for biochemical analysis of liver and kidney, while total food intake, feed efficiency ratio, and body weight gain were recorded.

#### 2.2.8 Histopathological analysis

Aflatoxins pre-carcinogenic impacts if the diet contains CGB or free were visualizing in rats-liver tissues. The tissues of each group were submerged individually by 10% formalin in sealed polypropylene container after the rats were slaughtered. Before microscopic examinations; tissues were dehydrated using graduated concentrations of ethyl alcohol then the known amount of xylene had been used. Liver tissues were cleaned using melton and prepared using sigma paraplast paraffin powder, sectioning into 5 μ slices utilizing a rotator microtome. Finally, it was stained with hematoxylin-eosin [31], for microscopic investigations (Axioskop 2 plus, Zeiss, Germany). The morphological evaluation was given for each group to explore the changes due to AFs presence, rats liver of groups fed on CGB-fortified diets were examined to explore its toxicity effect. Moreover, CGB was examined for aflatoxins reduction impact in orally AFs-administrated groups avoid the harmful impact in tissues. Randomly, numbers of liver sections were investigated for each group and the noted changes were recorded.

#### 2.2.9 Statistical evaluation

The obtained results were analyzed statistically using Analysis of Variance (One way ANOVA) using SPSS 16.0 as reported by McClave and Benson [32].

## 3 Results and discussions

### 3.1 Total antioxidants, phenolics, and flavonoids, content

Bioactive molecules play a good function in safety enhancement. Total phenolics and flavonoids content of fresh and dried CGB were determined. Table 1 indicates that; total phenolics of CGB in fresh and powder were recorded at 19.81 and 86.74 mg GAE/100 g, respectively. However, total flavonoids content showed an increasing values from 54.36 to 221.37 mg Cat/100 g by the drying process. Antioxidant potency of CGB powder was higher compared to the fresh fruits using three different antioxidant assays. Thus, it could support the function for avoiding oxidative stress caused by harmful substances or free radicals.

**Table 1:** Total phenolics, total flavonoids and antioxidant of fresh and dried cape-golden berry.

Sample	Total Phenol (mg GAE/100 g)	Total flavonoid content	ABTS (mgTE/g)	DPPH (mgTE/g)	FRAP (mgTE/g)
Fresh Cape- goldenberry	19.81 ± 1.46^b^	54.36 ± 1.36^b^	0.12 ± 0.06^b^	0.11 ± 0.02^b^	0.12 ± 0.08^b^
Dried Cape- goldenberry	86.74 ± 1.28^a^	221.37 ± 2.65^a^	3.77 ± 0.11^a^	4.65 ± 0.18^a^	3.81 ± 0.27^a^

● Data expressed as means ± SD.

● The data in the same column shared the superscriptions had no significant differences (*P* > 0.05).

### 3.2 Cape-goldenberry fortification impact on aflatoxin toxicity on rats’ feed capacity

An experiment was designed to evaluate the anti-toxicity of CGB against AFs. The results declared highly induces of final weight, body weight gain, and food efficiency of groups that orally administrated by AFs (G3 and G4), insertion of CGB powder in diets of AFs-treated rats showed less toxicity impacts on rats. Contaminated diets in the presence of CGB (G5 and G6) showed results close to the control (Table 2).

**Table 2:** Effect of diet-fortification using Cape-goldenberry on aflatoxin toxicity of in rats.

Parameters	Initial weight (g)	Final weight (g)	Weight gain (g)	Food intakes (g/d)	Food efficiency	ADG (g)	Relative WG %
Control (-) (G1)	160 ± 3.3	215 ± 4.2^c^	55 ± 2.74^c^	17.23 ± 0.16^b^	0.092 ± 0.003^c^	1.57 ± 0.013^b^	34.37^c^
Control (+) CGB (G2)	159 ± 4.1	216 ± 3.7^c^	57 ± 2.33^c^	17.08 ± 0.28^b^	0.095 ± 0.002^c^	1.63 ± 0.021^b^	35.84^c^
AFB_1_ (G3)	163 ± 3.77	196 ± 2.4^a^	33 ± 4.79^a^	16.78 ± 0.17^a^	0.056 ± 0.005^a^	0.94 ± 0.017^a^	20.24^a^
AFG_1_ (G4)	161 ± 2.79	198 ± 2.2^a^	37 ± 3.54^a^	16.81 ± 0.11^a^	0.063 ± 0.008^a^	1.05 ± 0.026^a^	22.98^a^
CGB + AFB_1_ (G5)	160 ± 5.11	204 ± 1.4^b^	44 ± 2.63^b^	17.61 ± 0.23^b^	0.071 ± 0.002^b^	1.26 ± 0.022^b^	27.5^b^
CGB + AFG_1_ (G6)	161 ± 3.22	206 ± 1.9^b^	45 ± 2.41^b^	17.56 ± 0.0.14^b^	0.073 ± 0.004^b^	1.29 ± 0.016^b^	27.95^b^

● Aflatoxins doses applied at 850 ng/kg body weight/day; g: means gram; d: means day, WG: weight gain, ADG: average daily gain.

● Data expressed as means ± SD; (n = 3; *P* > 0.05). ; The data in the same column shared the superscriptions had no significant differences.

● Control (+) diet contains cape-goldenberry dried powder at 20% -; CGB: Cape-goldenberry.

### 3.3 Cape-golden berry fortified diets impact on complete blood picture of rats

The blood components of experimental rats were evaluated for both the control and treatments. The data in Table 3 show the complete blood picture of rats included hemoglobin (HB), red blood cells (RBCs), white blood cells (WBCs), hematocrits (Hct), platelets (Plt), and two indications of iron in blood (TIBC and serum iron). Again, after the break-down the impact of AFB_1_ and AFG_1_ reflected on these parameters, The CGB exhibited good impacts correcting the toxicity influence. Nonetheless, a great iron reduction has happened due to AFs presence when it was evaluated as total iron binding capacity (TIBC) or for ashes (Inductively Coupled Plasma Atomic Emission Spectroscopy, ICP-MS). The fortification of diet by CGB showed improvements close to the control if AFs-treated rats were got it.

**Table 3:** Blood picture and iron changes for rats fed on diet supplemented by cape-goldenberry with/without aflatoxins orally administrated.

Parameters	control negative (G1)	CGB (G2)	AFB_1_ (G3)	AFG_1_ (G4)	CGB + AFB_1_ (G5)	CGB + AFG_1_ (G6)
HB (g/dL)	12.96 ± 0.14^b^	12.99 ± 0.19^b^	9.13 ± 0.61^a^	9.82 ± 0.48^a^	11.29 ± 0.33^b^	11.58 ± 0.46^b^
RBCs (10^6^/mm^3^)	7.62 ± 0.22^b^	7.7 ± 0.37^b^	4.6 ± 0.41^a^	5.12 ± 0.37^a^	7.34 ± 0.29^b^	7.4 ± 0.12^b^
WBCs (10^3^/mm^3^)	7.9 ± 0.31^c^	8.02 ± 0.26^c^	13.6 ± 0.52^a^	12.7 ± 0.45^a^	9.1 ± 0.36^b^	8.6 ± 0.24^b^
Hct (%)	36.2 ± 2.6^c^	37.4 ± 2.2^c^	53.7 ± 1.9^a^	47.6 ± 1.7^a^	44.6 ± 1.1^b^	41.9 ± 1.3^b^
Plt (10^3^/mm^3^)	844.8 ± 22.6^c^	847.2 ± 18.4^c^	497.4 ± 850^a^	528.5 ± 34^a^	767.1 ± 25^b^	801 ± 29^b^
TIBC (μg/dL)	446.2 ± 9.8^c^	443.3 ± 11.2^c^	645.9 ± 19.5^a^	652.4 ± 16.3^a^	508.1 ± 8.4^b^	515.6 ± 8.3^b^
Iron (ppm)	3.48 ± 0.07^c^	3.65 ± 0.05^c^	1.89 ± 0.07^a^	1.96 ± 0.08^a^	2.49 ± 0.11^b^	2.85 ± 0.09^b^

● Data expressed as means ± SD; (n = 3; *P* > 0.05); CGB: Cape-golden berry.

● TICB: Total iron binding capacity; HB: hemoglobin; RBCs: red blood cells; WBCs: white blood cells; Hct: hematocrits; Plt: platelets.

● The data in the same raw shared the superscriptions had no significant differences.

● Rats of aflatoxins treated groups were orally administrated by dose of 850 ng/kg body weight/day.

### 3.4 Biochemical parameters changes

**Fig. 1:**
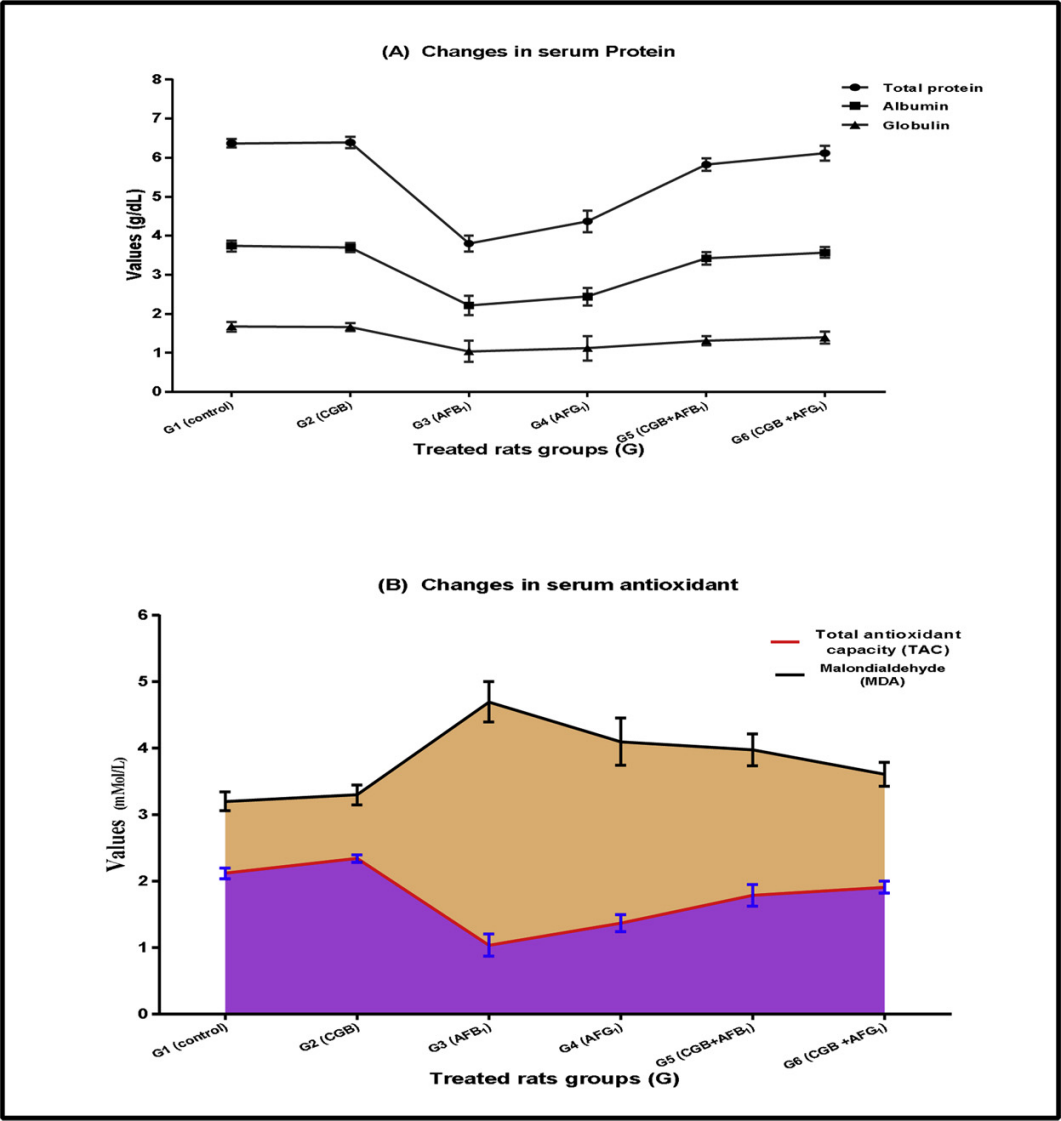
effect of Cap golden berry to limit serum proteins and serum antioxidant changes in aflatoxins-treated rats. ● Rats of aflatoxins treated groups were orally administrated by dose of 850 ng/kg body weight/day.

The CGB-diets were examined to reveal their functions as a corrective action that minimized pre-carcinogenic impacts of orally administrated AFs during the experiment. Table 4 and Fig. 1 showed major changes in AFs-rats serum contents of TP, ALB, GLB, LDL, HDL, TG, and TAA, particularly in the presence of AFs (G3 and G4). These changes were conjugated with AFs presence. Otherwise; insertion of CGB powder in rats-diet which orally administrated by AFs showed limited effects on rats’ biochemical parameters during the experimental period, this gives an evidence for CGB impact as AFs-inhibitor if the diet was contaminated (G5 and G6).

**Table 4:** Changes in rats’ serum lipids profile fed on Cape-goldenberry (with/without orally aflatoxins administration).

Parameters	control negative (G1)	CGB (G2)	AFB_1_ (G3)	AFG_1_ (G4)	CGB + AFB_1_ (G5)	CGB + AFG_1_ (G6)
CHL (mg/dL)	88.37 ± 7.1^c^	90.61 ± 4.9^c^	73.4 ± 3.1^a^	77.4 ± 2.4^a^	80.1 ± 3.2^b^	81.8 ± 2.5^b^
TG (mg/dL)	85.1 ± 1.73^d^	85.7 ± 2.42^d^	184.5 ± 3.61^a^	163.1 ± 4.45^a^	119.03 ± 3.42^b^	105.1 ± 3.81^c^
LDL-c (mg/dL)	55.4 ± 3.1^d^	55.2 ± 4.2^d^	102.1 ± 3.4^a^	91.6 ± 3.2^a^	73.4 ± 2.1^b^	62.1 ± 3.7^c^
HDL-c (mg/dL)	32.5 ± 1.3^d^	32.3 ± 1.5^d^	89.7 ± 2.3^a^	86.4 ± 4.1^a^	43.9 ± 3.7^b^	39.6 ± 3.3^c^
VLDL-c (mg/dL)	17.08 ± 1.64^d^	17.14 ± 1.73^d^	36.9 ± 1.72^a^	33.62 ± 1.89^a^	23.8 ± 1.68^b^	21.02 ± 1.76^c^

● CHL: cholesterol; TG: triglycerides; LDL-c: low-density lipoproteins; HDL-c: high-density lipoproteins; VLDL-c: very low-density lipoproteins.

● Data expressed as means ± SD; (n = 3, *P*>0.05) –; CGB: Cape-golden berry.

● Rats of aflatoxins treated groups were orally administrated by dose of 850 ng/kg body weight/day.

● The data in the same raw shared the superscriptions had no significant differences.

### 3.5 Effect of Cape-golden berry on enzymes in liver

Notwithstanding, liver enzymes were estimated in positive groups which oral injected by AFB_1_ and AFG_1_ compared to the control treatment. The results indicate a significant changes with high values, the toxicity impact of AFs drop-down the values of super oxidase dismutase, catalase, and glutathione S-transferease (Table 5). The values of lipid peroxidation represented by malondialdehyde showed elevation in AFs-treatment rats’ serum. It was obvious that; the AF-injected rats which fed a diet contains CGB recorded an improvement in enzymes value as compared to the control-recorded values. These also considered a guide to conclude that CGB reflect a reducing impact against AFs toxicity in the biological tissues.

**Table 5:** Changes in liver enzymes of rats fed cape-golden berry (with/without orally aflatoxins administration).

Parameters	control negative (G1)	CGB (G2)	AFB_1_ (G3)	AFG_1_ (G4)	CGB + AFB_1_ (G5)	CGB + AFG_1_ (G6)
SOD (m mol/L)	24.1 ± 3.1^d^	24.4 ± 2.3^d^	14.7 ± 4.2^a^	16.5 ± 4.1^b^	20.9 ± 1.3^c^	21.3 ± 2.2^c^
GST (μg/mg)	12.3 ± 1.51^c^	12.2 ± 1.73^c^	11.7 ± 1.1^a^	11.8 ± 2.0^a^	12.3 ± 1.2^b^	12.1 ± 2.3^b^
Catalase (μg/mg)	50.4 ± 4.23^d^	50.2 ± 5.34^d^	46.6 ± 4.3^a^	48.5 ± 6.7^b^	50.2 ± 4.1^c^	50.1 ± 3.6^c^
MDA (m mol/L)	0.55 ± 0.04^e^	0.54 ± 0.02^e^	0.94 ± 0.12^a^	0.87 ± 0.04^b^	0.71 ± 0.09^c^	0.64 ± 0.06^d^

● SOD: super oxidase dismutase; GST: glutathione S transferease; MDA: malondialdehyde.

● Oral administration of aflatoxin B_1_ or Aflatoxin G_1_ adjusted at 850 ng/kg body weight/day.

● Data expressed as means ± SD; (n = 3; *P* > 0.05); CGB: Cape-golden berry.

● The data in the same raw shared the superscriptions had no significant differences.

### 3.6 CGB reduction-impact on Liver and Kidney functions changes by aflatoxins

**Fig. 2:**
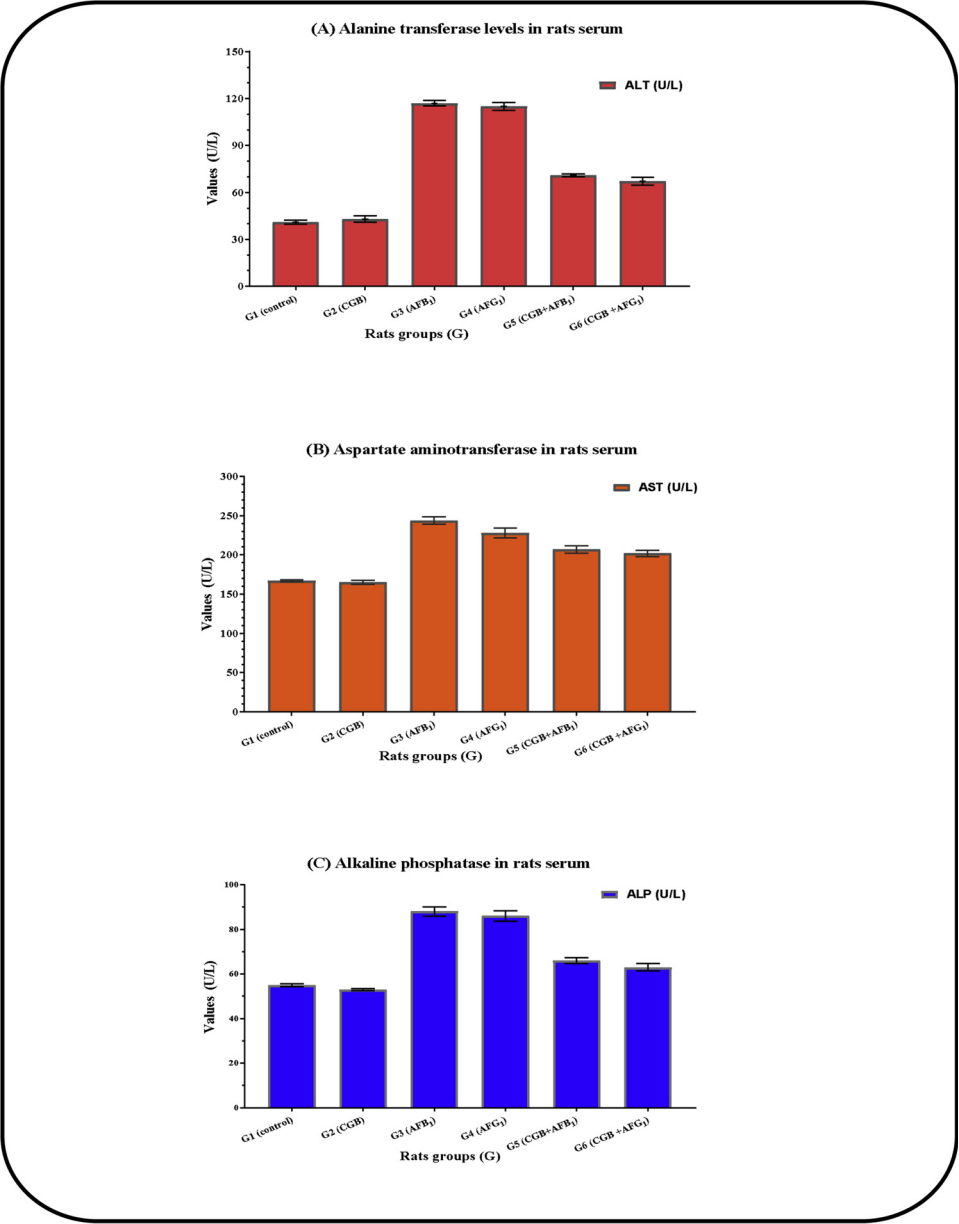
Liver functions of rats fed on cape-golden berry diet with/without aflatoxins orally administrated. ● Data expressed as means ± SD –; (n = 3; P = 0.05) –; CGB: Cape-golden berry. ● Oral administration of aflatoxin B_1_ or Aflatoxin G_1_ adjusted at 850 ng/kg body weight/day.

Due to the presence of AFB_1_ and AFG_1_ in rate serum, great changes were recorded in ALT, and AST that reflect the changes in liver functions, while changes were also noted for ALP values which reflected the marker for tumor occurrence (Fig. 2). Urea, uric acid, and creatinine were evaluated to explore the kidney function efficiency (Table 6**)**. These changes seemed to be lower if the CGB powder was supplemented to AFs-rats diet. Enhancement recorded could be correlated to the active components and oxidation-reduction potentials connected with the CGB presence in diets which may induced a breakdown of AFs toxicity.

**Table 6:** Kidney functions of rats fed on cape-goldenberry diet with/without aflatoxins orally administrated.

Parameters	control negative (G1)	CGB (G2)	AFB_1_ (G3)	AFG_1_ (G4)	CGB + AFB_1_ (G5)	CGB + AFG_1_ (G6)
Urea (mg/dL)	6.91 ± 0.04^b^	7.13 ± 0.06^b^	9.41 ± 0.28^a^	8.94 ± 0.34^a^	7.23 ± 0.09^b^	7.31 ± 0.06^b^
Uric acid (mg/dL)	2.31 ± 0.02^b^	2.26 ± 0.02^b^	5.63 ± 0.17^a^	5.34 ± 0.11^a^	2.71 ± 0.08^b^	2.43 ± 0.05^b^
Creatinine (mg/dL)	0.96 ± 0.01^b^	0.99 ± 0.04^b^	1.32 ± 0.31^a^	1.21 ± 0.19^a^	1.07 ± 0.05^b^	1.03 ± 0.06^b^

● Data expressed as means ± SD –; (n = 3, *P*>0.05) –; CGB: Cape-goldenberry.

● Oral administration of aflatoxin B_1_ or Aflatoxin G_1_ adjusted at 850 ng/kg body weight/day.

● The data in the same raw shared the superscriptions had no significant differences.

### 3.7 Aflatoxins effect and a corrective action of CGB powder on liver tissues of rats

**Fig. 3:**
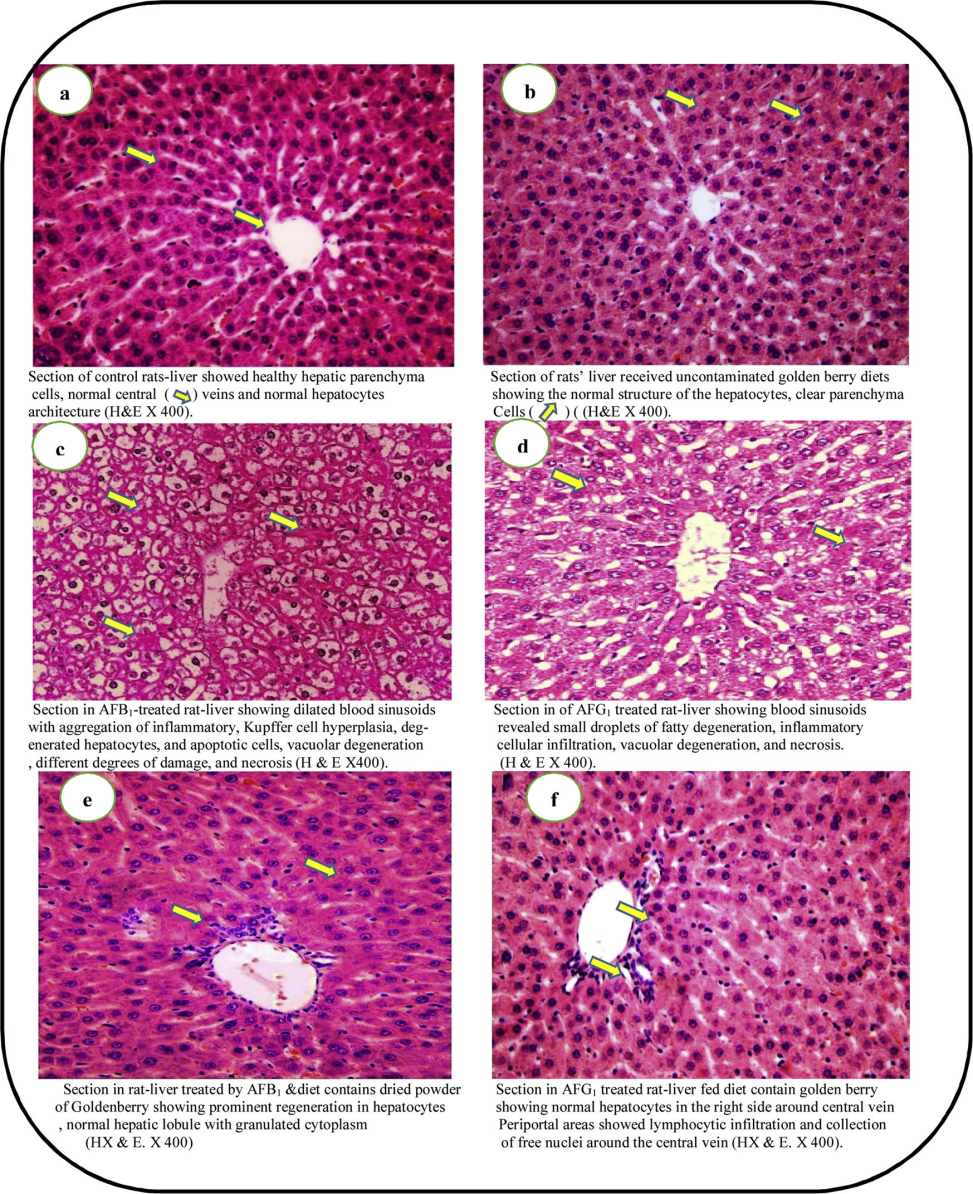
Liver tissues of rats fed diets contained a dried golden berry powder to avoided aflatoxins pre-carcinogenic impacts. ● Rats of aflatoxins treated groups were orally administrated by dose of 850 ng/kg body weight/day.

Aflatoxins effect and a corrective action of CGB powder on liver tissues of rats were evaluated to explain the ability of CGB in a corrective action against the toxicity effects. Liver tissues of rates were captured using Nikon microscope with camera and the resulted captures were presented in Fig. 3. A normal liver tissue structure of rats was showed without any damages recorded Fig. 3a. The hepatic lobules are formed a rows of polyhedral hepatocytes containing a nuclei and abundant cytoplasm. There are blood sinusoids in – between the hepatocytes separated from them by endothelial cells. A healthy hepatic parenchyma cell tissues, central veins, and normal hepatocytes architecture could be shown at the magnification power (H&E X 400). The walls of the sinusoids contain phagocytic irregular cells with multiple processes known as Von Kupffer. The sinusoids run radially, converging at the centre of the hepatic lobule to form the central or centrolobular vein. Fig. 3b shows the effect of oral administration of CGB on liver tissue structure of rats. The tissues showed normal structure of the hepatocytes and a clear parenchyma cells. Fig. 3c and d show the effect of four weeks oral administration, at 850 ng/kg body weight/day of AFB_1_ and AFG_1_, respectively, for each rat on its liver tissue. The liver showed macroviscular fatty change and hydropic degeneration (Fig. 3c). The AFB_1_ treated rat showed dilated blood sinusoids with aggregation of inflammatory, Kupffer cell hyperplasia, degenerated hepatocytes, and apoptotic cells, vacuolar degeneration, different degrees of damage, and necrosis. In rats which were treated by AFG_1_, congested hepatic sinusoids and mild lymphocyte infiltration was noted. AFG_1_ treated rat showed blood sinusoids revealed small droplets of fatty degeneration, inflammatory cellular infiltration, vacuolar degeneration, and necrosis (Fig. 3d).

Fig. 3e and f point out the effect of oral administration of CGB–AFB_1_ and CGB-AFG_1_ toxins for five weeks on rats' liver. In most rats, liver hepatocytes appeared to be almost normal similar to the control (Fig. 3f). In few rats there was some congestion and dilation of the portal area with micro-vesicular fatty change, a few areas of hydropic degeneration and free nucleus were noted around the central vein (Fig. 3e). Results pointed out that CGB recorded a corrective action for the toxicity which induced incase that AFs were present in the diets. The results also give an indication of the alleviated effect of CGB against the harmful materials which could contaminate the diets along with the improvement that recorded on liver tissues.

Aflatoxins and their biological metabolites cause a chain of exceptional reaction leads to oxidative stress in the liver. This stress plays a principal function in aflatoxicosis diseases. The most famous AFs is AFB_1_ which is related to mutagenic contaminants of food causing potent hepatocarcinogens for human and animal tissues. During the metabolic pathway of AFB_1_, an increment of free radicals production and lipid peroxides occurs, this finally leads to cell damages [33]. Aflatoxins, by reached the liver organ principally pass through a biotransformation process. This transformation is classified by two stages: the first stage covered both oxidative, reductive, and hydrolytic reactions. In that stage; an activation or fragmentation could happen for the compound structure gives more active/toxic substances. This stage inclusive regularly in the enzymes system of cytochrome P450. Moreover, this stage considered the fundamental structure for the second stage of reactions which is including the compound conjugation, particularly to the DNA strands. Whereas the second stage of AFs-reactions in the liver leads both to detoxification and formation of biochemical lesions [34].

The expanded exposure for various dietary carcinogens, particularly the aflatoxins, does consider a principal factor driving to chronic infections including B or C hepatitis viruses. While the total removal of aflatoxins is not quite possible, various bioactives are nominating for aflatoxin degradation. Chemo-prevention realized since the natural chemical factors utilization for converting or overcoming the carcinogenic progress of cancer. Recently, remarkable articles gave an informative and reviews concerning the chemo-preventive effects of phytochemicals against hepatocarcinogenesis, including the polyphenols and flavonoids [35,36].

As outcomes of this investigation, this is a proper detoxification method for food and food products, since CGB could be a regular part of a food process. These results proved that, the use of CGB for toxin effect alleviated the harmful induced according to toxin presence. This was proved by the feeding experiment in which feeding the CGB to an orally-toxins-treated rats showed almost normal liver tissues. Therefore, screening of different natural plant materials that could include a high content of bioactive ingredients for its impact on toxin alleviated is recommended to find new high rate toxin reduction sources. Moreover, the use of CGB adaptation method tried in this work would help in enhancing the rate of alleviated of a particular natural plant material needed for a particular food processing.

In the present study; the protective impact of CGB dry powder against pre-carcinogenic AFB_1_ and toxicity of AFG_1_ was examined in male rats as a biological experimental model. Aflatoxins, particularly AFB_1_, are a major risk counter the healthful parameters and causing several issues for humans and animals. Because of the consideration that AFB_1_ is pre-carcinogenic compound [16], also it was found excreting by toxigenic fungi on food commodities [18,23] at several stages (pre-harvest; post-harvest; transportation; storage; handling). In this respect, a need for novel strategies avoiding mycotoxin health hazard of contaminated diets is requesting. The CGB provides a modern integrated source of bioactive materials and antioxidants [1,4,5,10]. The CGB analysis showed a presence of various active substances either in fresh or dried fruit (Table 1), these ingredients could explain their activity against AFs-oxidative stress reduction [37] recorded for rats’ biochemical parameters. Regarding AFs oral-administrated to rat groups (G3 to G6), physical properties were found to changes. Decreases of final weight, food efficiency, and relative weight gain were traced. These results agree with the previous studies which declared AFs impacts on rats food intakes, food efficiency, and relative weight gain [38,39]. However, the presence of CGB in feeding enhances these parameters if it was supported in diets (G5 and G6).

Blood parameters reported changes in case of diets contaminated by AFs [40,41]. The HB, RBCs, WBCs, Hct, and Plt showed a dropped in the presence of only AFs in rats’ biological system. The enhancement was recorded for blood parameters of AF-administrated rats in case that CGB had been presented in diets. This also happened for the iron level of blood which actually estimated by the TIBC. Concerning the serum lipid data, AFs is known to occurr risks due to changes happened CHL and TG values being [16,42,43]. In the G3 (AFB_1_ treated) and G4 (AFG_1_ treated) major changes of CHL, TG, LDL-c, and HDL-c values occurred compared to the control. These values showed turn close again to the control by diet CGB supplementation (Table 4). The vLDL-c values were referred to as risk occurrence in case that AFB_1_ or AFG_1_ orally administration to rats of G3 and G4. This risk factor came-down by CGB addition in diet contents (G5 and G6). On the other hand; reduction of protein and its fractions by AFs contamination of rat-diets were observed [44]. The data of the present study showed the TP, ALB, and GLB enhancement in treated rats (AFB_1_ or AFG_1_) after they fed CGB-diets (Fig. 1). Again, liver and kidney functions side to other liver enzymes reported were influencedby AFs presence in rats’ biological system [[44], [45], [46]]. Moreover, the histological studies of rats’ tissues recorded good influences of CGB when it was added to AFs-rats diets (Fig. 3e–f).

The SOD and CAT are responsible for the detoxification in a cellular system. The SOD is the primary defense against oxygen free radicals in the cells through the catalyzing of superoxide radicals. The AFs causes depletion of SOD and CAT activity, as well as TAC and GSH contents, in all examined organs. The present investigation showed a significant loss for antioxidant enzyme activities in livers of AFs-rats. The marked reduction in SOD activity suggests the inactivation of antioxidant enzymes due to risen superoxide radical production [47]. The marked reduction of GST, SOD, and CAT levels of AFs-rats associated with lipid peroxides accumulation that increase during hepatotoxicity.

The changes of AST, ALT, and ALP are significant parameters considered to be the potential biomarkers for AF’s-induced oxidative stress or pre carcinogenic marker [44]. During the experiment; elevation of serum AST, ALT, and ALP, with ALT and AST-decreases in rats' liver has been recorded. These due to hepatocytes damage resulted by AFs-exposure causing hepatic dysfunction and subsequent leakage of these enzymes from the neoplastic cell into circulation, this finding supported by the earlier one [48]. Also, due to the possible effect of AFs on remote tissue leading to leakage of enzymes released into the blood [49]. Furthermore, alkaline phosphatase (ALP), as the tumor marker, was changed in AFs-rats. This observation for ALP-increase in rats previously linked to gene-altered [50]. In the present study, the AFs-treated group suffered from severe oxidative stress in different organs, achieved by elevation of MDA level and depletion of antioxidant enzymes. This due to the conversion of cellular poly-unsaturated fatty acids to the toxic product inhibits cellular protective enzymes [51]. Hepatotoxicity caused by AFs generally reflects the instability of liver metabolism associated with free radicals species generation, leads to oxidative stress and alterations in antioxidant defense mechanisms.

In recent years, there has been growing interest in natural bioactive compounds which have a therapeutic impact and chemoprotective properties against various diseases including hepatotoxic and carcinogenic [52,53]. The benefits of CGB are associated with their consumption due to its nutritional and health benefits. The results showed enormous impacts of CGB supplementation alleviating the oxidative stress. This attributed to CGB antioxidant scavengers impact causing enhancement cellular defense functions. This was occurred through modulating the alteration in GST content and antioxidant enzymes activity. The CGB contains different compounds included phytochemicals, hydroxyl-withanolide, withanolides, phygrine, kaempferol, and quercetin [54,55]. These compounds have a strong antioxidant property and prevent oxidative damage to liver microsomes and hepatocytes which could be caused by harmful compounds like aflatoxins [16,56]. Moreover, CGB has been stated rich in polyunsaturated fatty acids, vitamins, phytosterols, and essential minerals which gives CGB medicinal properties [3].

In the light of these results, CGB could record a better impact against the pre carcinogenic compounds particularly AFB_1_. The CGB efficiency to avoid the carcinogenic materials represented by several parameters including serum lipids, serum proteins, blood parameters, liver and kidney functions. The enhancement of CGB against AFB_1_ and AFG_1_ impacts in blood, serum, and tissues gave evidence on its effect as an avoiding pre-carcinogens.

## 4 Conclusion

Aflatoxins are great harmful compounds with pre-carcingenic impact. It could contaminate various types of food and feed materials included cereals. Fruit has bioactive substances in edible or non-edible parts. The CGB was chosen for its major bioactive molecules, it was dried under vacuum, milled, and sieved. The CBG impact against AFs harmful was evaluated using experimental rats. The blood and biochemical parameters, liver and kidney functions, liver enzymes, also serum lipids were estimated. Liver tissues were investigated for AFs, CGB, and AFs-CGB impacts. Results declared improvement of food efficiency, body weight gain, and blood parameters for AFs-rats by CGB diet-fortification. Moreover, liver (enzymes and tissues) showed enhancement if diet of AFs-rats contains CGB powder.
